# Primary subacute hematogenous osteomyelitis of navicular bone: A rare case report in 7-year-old child

**DOI:** 10.1016/j.amsu.2021.102911

**Published:** 2021-10-05

**Authors:** Zairi Mohamed, Ahmed Msakni, Rim Boussetta, Ahmed Amin Mohseni, Mohamed Nabil Nessib

**Affiliations:** Faculty of Medicine of Tunis, Department of Pediatric Orthopedic Surgery, Bechir Hamza Children's Hospital, Tunis, Tunisia

**Keywords:** Children, Primary subacute hematogenous osteomyelitis, Navicular bone, Foot

## Abstract

**Introduction and importance:**

Navicular bone location of primary subacute hematogenous osteomyelitis uncommon. There are few cases reported in literature. We aim to describe the clinico-radiological features of primary subacute hematogenous osteomyelitis of the navicular bone in a 7-year-old child, to explain our management of this rare disease and demonstrate that medical treatment without surgery is enough.

**Case presentation:**

A 7-year-old child presented to emergency department. His chief complaint was fever, left limping and foot pain. The positive examination features of were painful palpation of the dorsal side of the foot and a swelling of the homolateral ankle. The erythrocyte sedimentation rate was high. The x-ray revealed a lytic lesion of the left navicular bone. The MRI findings led to the diagnosis of subacute osteomyelitis. Pain relief and normalization of inflammatory markers were obtained after 8 weeks of antibiotic therapy. Complete radiological healing was obtained after 9 months. One year after treatment, the patient was able to practice sports as previously.

**Clinical discussion:**

Subacute osteomyelitis of the navicular bone in pediatric population is a rare condition. This case shows the importance of early diagnosis thanks to MRI findings and appropriate antibiotic therapy based on the endemic bacteriological profile.

**Conclusion:**

The navicular bone may develop primary subacute osteomyelitis in immunocompetent child. Early diagnosis is important for prescribing effective conservative treatment.

## Highlights

• Subacute osteomyelitis of the navicular bone is an etiology of lameness in children.
• The penumbra sign on MRI is strongly suggestive of the infectious origin of the bone involvement.
• Medical treatment without surgery can lead to a cure.
• Antibiotic therapy should be sufficiently prolonged for at least 3 months.

## 1 Introduction

Primary subacute hematogenous osteomyelitis is often located at the metaphysis of long bones. Its location in the foot small bones is uncommon. We present a rare case of primary subacute hematogenous osteomyelitis of the navicular bone in a 7-year-old immunocompetent child, treated medically without surgery. Few cases are reported in the literature [1]. There is no consensus for therapeutic management. Through this case, we will report our experience in order to demonstrate that medical treatment without surgery may be enough.

This case report has been reported in line with the SCARE Criteria [2] Agha RA, Franchi T, Sohrabi C, Mathew G, Kerwan A; SCARE Group. The SCARE 2020 Guideline: Updating Consensus Surgical CAse REport (SCARE) Guidelines Int J Surg. 2020; 84:226–230.

## 2 Case presentation

**Fig. 1:**
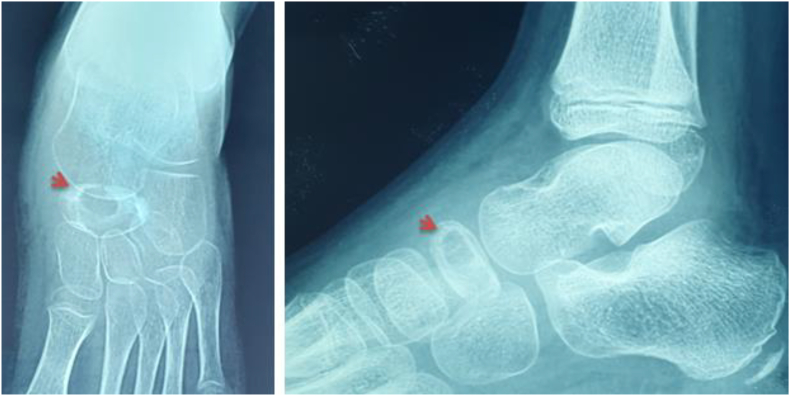
Oval osteolysis of the body of the navicular bone with condensation of the peripheral border (Arrow) showed on the x-ray of the foot.

**Fig. 2:**
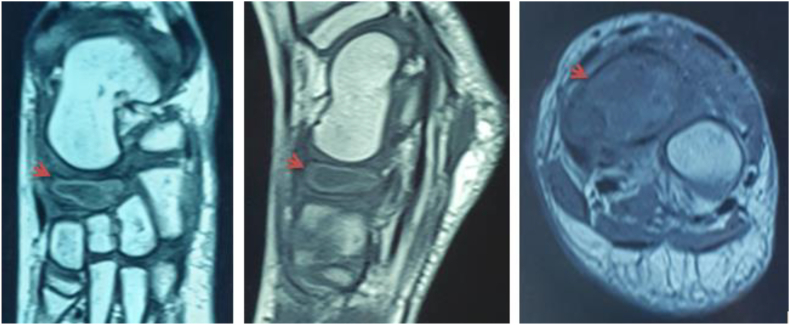
Unenhanced T1-weighted MRI sequences: T1 hypointense of the spongy bone of the navicular bone (arrows) on the coronal, sagittal and axial slices showing the “penumbra sign” described as perilesional lining of higher signal intensity, with a central hypo intense content.

**Fig. 3:**
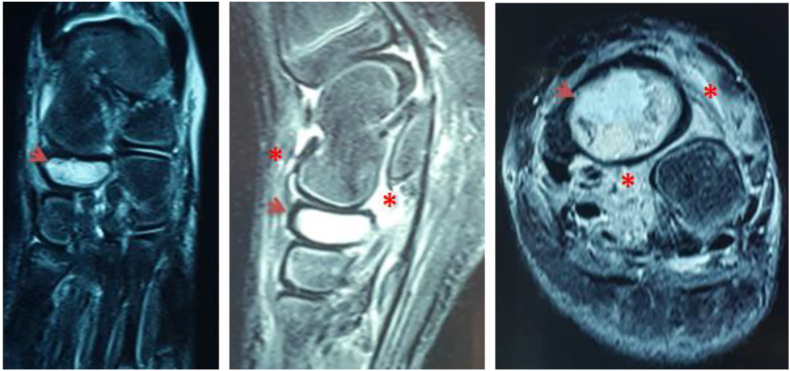
T2-weighted MRI sequences: Intense T2 hypersignal of the spongy bone of the navicular bone (Arrows) on the coronal, sagittal and axial slices associated with an inflammatory reaction in the perinavicular tissue (Asterisk).

**Fig. 4:**
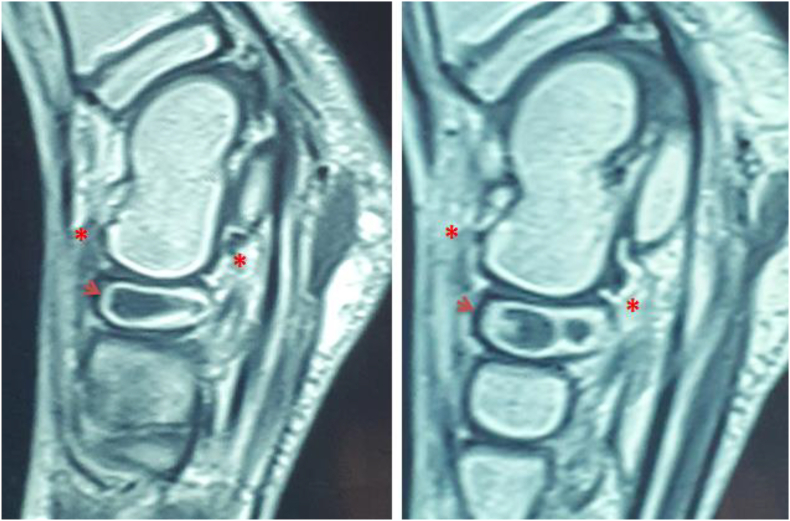
Enhanced T1-weighted MRI sequences: Peripheral T1 hypersignal enhancement of the navicular bone (Arrows) and perinavicular soft tissue (Asterisk).

We report the case of a 7-year-old child, with no medical history. He presented to the pediatric orthopedic emergency department for lameness that has been evolving for a month and had worsened 3 days ago. The parents reported a history of a trauma to the left foot a month ago. Clinical examination confirmed left lameness, fever and a painful swelling of the back side of the foot without inflammatory signs. The ankle had painless full range of motion and there were no skin lesions. The x-ray of the foot showed an osteolytic lesion of the body of the navicular bone without cortical rupture (Fig. 1). The blood count showed an increased level of white blood cells with a value of 10970 elements/ml and neutrophils at 4270 elements/ml and a normal C-reactive protein = 1 mg/L. The erythrocyte sedimentation rate was increased to 25 mm for the 1st hour. The MRI of the foot was performed to better the etiological assessments. It objectified signal abnormalities of the navicular bone: hypointense in T1 sequence (Fig. 2) and in clear hypersignal in T2 sequence (Fig. 3), enhanced peripherally after injection of Gadolinium with a discreet spontaneous hypersignal ring in T1 evoking the “Penumbra” Sign (Fig. 4). It was associated with an inflammatory reaction of perinavicular soft tissues without collection and of the ankle and talonavicular joint synovitis. In the presence of these clinical, biological, and radiological features, the diagnosis of primary subacute hematogenous osteomyelitis of navicular bone was retained. The blood culture was negative.

**Fig. 5:**
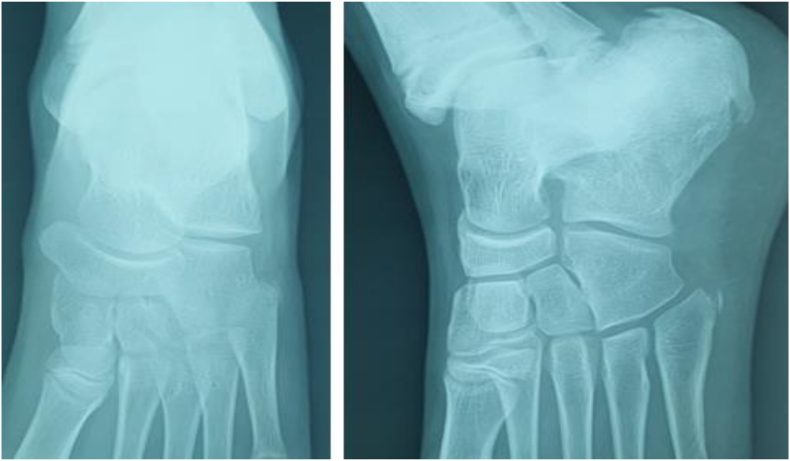
X-ray of the navicular bone at 5 years follow-up: no sequelae.

The patient undertook antibiotic therapy: Clavulanic acid/Amoxicillin 100mg/Kg/day or 800 mg three times a day intravenously for one week and Gentamycin 4mg/Kg/day or 90 mg once a day as an infusion for 4 days. Then Clavulanic acid/Amoxicillin per os for a total duration of 4 months. In association with the medical treatment, a cast boot was put for 6 weeks. The pain relief was obtained 8 weeks after antibiotic therapy onset. Biological monitoring did not show any abnormalities. Radiological monitoring showed calcification of the bone gap after 3 months of treatment. The patient was able to practice sports as previously after 1 year. At 5 years follow-up, there was no sequelae in the navicular bone (Fig. 5).

## 3 Discussion

Subacute osteomyelitis can be difficult to diagnose because the characteristic signs and symptoms of acute infection are often absent [2].Subacute osteomyelitis of the navicular bone in an immunocompetent child is a very rare condition. It is difficult to determine the actual mainstream treatment of navicular bone osteomyelitis because cases are very rare [3]. Two potential pathways of osteomyelitis of the body of the navicular bone are worth discussing. Hematogenous contamination or contiguity contamination such as atopic dermatitis of the foot during Wiskott – Aldrich syndrome and cellulitis [1]. In subacute osteomyelitis, MRI has an essential role. It makes it possible to evoke the diagnosis by objectifying characteristic elements such as “Penumbra sign”, and to evaluate the extent of the lesions and to assess the effectiveness of the treatment [1,4]. Cakmak Celik et al. [4] have published a case report of osteomyelitis of the navicular bone explored by MRI in a immunocompetent child and the navicular bone abscess was surgically drained. Takahashi et al. [5] reported a correlation between a diminished edema-like bone marrow pattern on MRI and alleviation of symptoms in pediatric population. Kumahashi et al. [1] reported that after starting antibiotic therapy, blood patterns return to normal at one month, the pain disappears at three months, and the MRI signal abnormalities may persist for eight months. Moreover, MRI is the best non-invasive imaging modality to evaluate osteomyelitis. In fact, the combination of intramedullary patterns of decreased signal intensity on T1-weighted images is the most reliable sign to diagnose osteomyelitis [6]. The “Penumbra sign” in MRI is helpful in distinguishing between subacute osteomyelitis from other osseous lesions but it is not pathognomonic [7,8]. It can be identified on unenhanced T1-weighted spin echo images as a discrete peripheral zone of marginally higher signal intensity than the central bony abscess cavity and the surrounding lower signal intensity of the reactive new bone and edema [2].

In the current case, radiological healing was late according to the clinical one because the navicular bone is poorly vascularized. The duration of antibiotic therapy is not standardized. It ranged from two months for Cakmak Celik [4] et al. to eight months for Kumahashi et al. [1] because their patients were not immunocompetent, and the assessment was based on the normalization of MRI features [1]. In children, the optimal duration of antibiotic therapy is still unclear. Because of the poor vascularization of the navicular bone there is a low concentration of antibiotics unlike metaphysis of long bones, that's why the antibiotic therapy should be undertook more than usual. In the absence of identification of the causal germ, the effectiveness of antibiotics is judged by the pain relief, the normalization of the erythrocyte sedimentation rate and the disappearance of signal abnormalities in MRI.

Surgical treatment in subacute osteomyelitis has not been unanimous due to the low number of sporadic cases. Cakmak Celik et al. [4] have reported surgical treatment. Kumahashi et al. [1], reported conservative treatment with antibiotics in subacute osteomyelitis of the navicular bone. In our case, there was no abscess. Hence, we chose a conservative treatment with antibiotics.

The most common and reported pathogen germ related to subacute osteomyelitis in children has is staphylococcus aureus [9]. The juvenile form affects children over 4 years old, and staphylococcus aureus seems to be the main germ [9]. However, recently, other bacteria such as Salmonella [4], Kingella kingae [9] and Morganella morganii [10] have been reported.

The immobilization of the foot in a plaster is an important part of the treatment. In fact, it allows bone to heal and to avoid pathological fractures [1,4].

## 4 Conclusion

The navicular bone may develop primary subacute osteomyelitis in immunocompetent child. Early diagnosis is important for the onset of effective conservative treatment. Surgery is not necessary in the absence of a soft tissue abscess. Prolonged antibiotic treatment and the immobilization of the foot are the mainstream treatment.

## Data availability

All data is available to readers.

## Ethical approval

None.

## Funding

None.

## Author contribution

Mohamed Zairi Writing drafting the article. Ahmed Msakni conception and design, revising it critically. Rim Boussetta revising it critically for important intellectual content. Ahmed Amin Mohseni revising it critically. Mohamed Nabil Nessib final approval of the version to be published.

## Consent

Written informed consent was obtained from the patient for publication of this case report and accompanying images. A copy of the written consent is available for review by the Editor-in-Chief of this journal on request.

## Registration of research studies

1. Name of the registry:

2. Unique Identifying number or registration ID:

3. Hyperlink to your specific registration (must be publicly accessible and will be checked):

## Guarantor

Mohamed Zairi.

## Provenance and peer review

Not commissioned, externally peer-reviewed.

## Declaration of competing interest

None.
